# Immune checkpoint landscape in CD4⁺ T cells stratifies HIV-infected individuals by clinical progression

**DOI:** 10.1007/s12026-026-09779-x

**Published:** 2026-04-29

**Authors:** Serena Spampinato, Grazia Scuderi, Michelino Di Rosa, Paolo Fagone, Giuseppe Nunnari

**Affiliations:** 1 Unit of Infectious Diseases, ARNAS “Garibaldi Nesima” Hospital, Department of Clinical and Experimental Medicine, University of Catania, Catania, 95122 Italy; 2 Department of Biomedical and Biotechnological Sciences, University of Catania, Catania, 95123 Italy

**Keywords:** Immune checkpoints, HIV, Elite controllers, T helper lymphocytes

## Abstract

Immune checkpoint (IC) pathways play a central role in modulating HIV-specific T cell responses and may influence the degree of viral control. Here, we performed a comparative transcriptomic analysis of CD4⁺ T cells from HIV-infected individuals classified as elite controllers (EC), viremic controllers (VC), and chronic progressors (CP), aiming to identify IC signatures associated with viral control. ECs exhibited distinct expression profiles, characterized by significant upregulation of *CEACAM1* and *CD274* (*PD-L1*), and downregulation of *CD200*, *TIGIT*, *CTLA4*, *BTLA*, and *ADGRG1* relative to CPs. VC samples displayed intermediate expression levels. Principal component analysis (PCA) of IC genes revealed clear separation between ECs and CPs, driven largely by the differential expression of *PDCD1*, *CTLA4*, *TIGIT*, and *CD28*. ECs were also enriched in effector memory and Th2 CD4⁺ T cell subsets, which correlated positively with *CEACAM1* and *PD-L1*, and inversely with *TIGIT* and *CTLA4*. Co-expression network analysis identified two distinct gene modules: one (M2) containing *CEACAM1* and *PD-L1*, enriched for interferon signaling and NF-κB pathways, and another (M4) comprising *TIGIT*, *CD200*, and *BTLA*, enriched for TCR signaling and metabolic processes. Finally, comparison of ECs, ART-naïve, and ART-treated individuals showed that pre-ART subjects displayed significantly elevated *ADGRG1* expression, which decreased following ART initiation, resembling the EC profile. These findings reveal checkpoint-related molecular signatures and cell subset compositions that stratify HIV-infected individuals by disease control phenotype, and highlight potential targets for immunomodulatory therapies aimed at achieving functional cure.

## Introduction

Human immunodeficiency virus (HIV) infection remains one of the most significant challenges in modern medicine due to its intricate ability to evade immune surveillance and establish latent reservoirs. These reservoirs persist even under effective antiretroviral therapy (ART), preventing complete viral eradication [1, 2]. While ART can suppress viral replication and significantly reduce AIDS-related morbidity and mortality, it does not constitute a cure [3–6]. The virus integrates into the genome of long-lived immune cells, particularly memory CD4⁺ T cells, and can rapidly reactivate upon treatment interruption [7, 8]. A hallmark of chronic HIV infection is persistent immune activation and the gradual deterioration of immune function, leading to a phenomenon known as T cell exhaustion [9]. Exhausted T cells exhibit impaired proliferative capacity, reduced cytokine production, and diminished cytotoxicity. This dysfunctional state is closely associated with increased expression of inhibitory receptors, known as immune checkpoint molecules [10, 11]. Among the most studied are PD-1 (Programmed cell death protein 1), TIM-3 (T cell immunoglobulin and mucin-domain containing-3), LAG-3 (Lymphocyte activation gene 3), and TIGIT (T cell immunoreceptor with Ig and ITIM domains), which have all been implicated in the dysregulation of antiviral immunity [12–19]. These immune checkpoints play essential roles in maintaining peripheral tolerance and limiting immune-mediated damage by dampening T cell responses. However, in chronic infections like HIV, their sustained expression contributes to immune exhaustion and facilitates viral persistence [20, 21]. Numerous studies have demonstrated that high expression levels of PD-1 and other checkpoints correlate with elevated viral loads, diminished HIV-specific T cell function, and a larger viral reservoir [22, 23]. Interestingly, not all HIV-positive individuals progress similarly. Subgroups such as elite controllers (ECs) and viremic controllers (VCs) can suppress viral replication in the absence of ART. Elite controllers, who represent less than 1% of HIV-infected individuals, maintain undetectable viral loads (< 50 copies/mL) without therapy for prolonged periods, largely due to robust and polyfunctional immune responses [24, 25]. Viremic controllers also spontaneously maintain low but detectable viremia (< 2000 copies/mL), albeit with less efficiency than ECs [26]. In contrast, chronic progressors (CPs) are characterized by persistent viremia and progressive immune decline in the absence of treatment [26, 27]. These distinct phenotypes provide a valuable model for studying natural viral control. In particular, differences in immune checkpoint expression may offer critical insights into mechanisms of viral containment. Lower expression of inhibitory receptors in ECs and VCs has been hypothesized to allow for more effective T cell responses, highlighting the potential of targeting these pathways for therapeutic benefit [26, 28, 29]. In this context, our study aimed to characterize the expression of 30 immune checkpoints from well-defined cohorts of ECs, VCs, and CPs. Our goal was to establish immunophenotypic signatures associated with varying degrees of viral control and to assess the correlation between checkpoint expression and disease progression. This analysis may ultimately inform strategies to enhance host immunity through immune checkpoint modulation. By comparing checkpoint expression across individuals with differing abilities to control HIV, novel targets for immunotherapeutic strategies can be designed to reverse exhaustion, reduce reservoirs, and ultimately contribute to a functional cure.

## Materials and methods

### Data acquisition

Transcriptomic data analyzed in this study were retrieved from the publicly available Gene Expression Omnibus (GEO) database. Specifically, we utilized the GSE128297 dataset [30], which includes gene expression profiles from purified CD4⁺ T cells isolated from HIV-infected individuals categorized as elite controllers (EC), viremic controllers (VC), chronic progressors (CP), as well as individuals sampled before and after initiation of antiretroviral therapy (pre-ART and post-ART, respectively). Chronic progressors (CP) were defined as individuals with plasma viral loads exceeding 5,000 HIV RNA copies/mL, while viremic controllers (VC) maintained viral loads between 50 and 5,000 copies/mL. Elite controllers (EC) were characterized by their ability to maintain viral loads below 50 copies/mL without the need for therapy. All participants classified as CP, VC, or EC were either antiretroviral treatment-naïve or had been off therapy for at least three months prior to sampling. Gene expression profiling was performed using the Affymetrix Human Clariom D Assay microarray platform. Further details regarding cohort characteristics and dataset generation are described in Morou et al., 2019 [30].

### Immune checkpoint expression profiling

A panel of 30 immune checkpoint genes was selected for analysis, encompassing classical inhibitory receptors (e.g., *PDCD1*, *CTLA4*, *TIGIT*, *LAG3*), co-stimulatory molecules (e.g., *CD86*, *CD80*, *TNFRSF14*), and immunoglobulin-like receptors (e.g., *LILRB4*, *SIGLEC10*). Gene expression values were extracted from the normalized dataset, and unsupervised hierarchical clustering along with heatmap visualization was performed using the Morpheus web-based platform (https://software.broadinstitute.org/morpheus). Differential expression analysis among clinical groups was carried out using the limma package in R, and multiple testing was corrected using the Benjamini–Hochberg method (adjusted p-value < 0.05 considered significant). Principal component analysis (PCA) was performed using the SRPlot online tool (https://www.bioinformatics.com.cn) to assess variance in immune checkpoint gene expression across the different clinical phenotypes. Biplots were generated to illustrate sample distribution and the contribution of individual genes to principal components, enabling the interpretation of group-specific clustering based on the orientation and magnitude of gene loadings.

### Immune profiling and correlation analysis

To estimate the relative abundance of CD4⁺ T cell subpopulations, transcriptomic deconvolution was performed using the xCell online tool (https://xcell.ucsf.edu/), which applies a gene signature-based approach to infer the enrichment of various immune and stromal cell types from bulk transcriptomic data. The resulting enrichment scores for CD4⁺ T cell subsets, including effector memory, central memory, Th1, and Th2 populations, were used for downstream correlation analysis. Spearman’s rank correlation was employed to assess the relationships between the abundance of specific CD4⁺ T cell subsets and the expression levels of selected immune checkpoint genes. Correlation matrices were generated using the SRPlot platform (https://www.bioinformatics.com.cn), and correlation coefficients (ρ) along with significance values were used to interpret the strength and direction of associations.

### Gene co-expression network analysis

Co-expression network analysis was performed using the CEMiTool R package, which automatically detects gene modules based on expression similarity across the dataset. The normalized expression matrix was used as input, and default parameters were applied for module detection and enrichment analysis. Modules containing selected immune checkpoint genes were identified and extracted for further characterization. These modules were subsequently analyzed using the MCODE algorithm within the Metascape platform (https://metascape.org) to identify densely connected subnetwork components. Functional annotations of MCODE clusters were automatically generated based on integrated pathway databases.

### Statistical analysis

Differential gene expression between clinical groups was assessed using the limma package in R, applying empirical Bayes moderation and adjusting for multiple testing with the Benjamini–Hochberg false discovery rate (FDR) correction; adjusted p-values < 0.05 were considered statistically significant. Spearman’s rank correlation was employed to evaluate associations between CD4⁺ T cell subpopulations and immune checkpoint gene expression. Principal component analysis (PCA) was used to explore global expression variation and group clustering. Correlation plots, PCA biplots, and bar graphs were generated using SRPlot (https://www.bioinformatics.com.cn) and GraphPad Prism. Functional enrichment analyses were based on hypergeometric tests as implemented in Metascape, with significance defined by FDR-adjusted p-values.

## Results

### Distinct immune checkpoint expression patterns stratify HIV-infected individuals by disease control phenotype

**Fig. 1:**
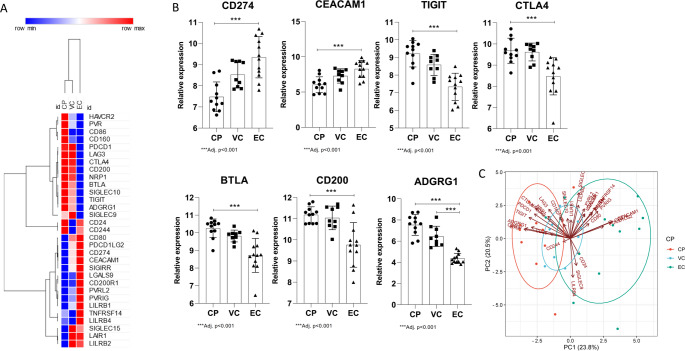
Immune checkpoint gene expression patterns stratify HIV clinical phenotypes. **A** Heatmap showing expression of selected immune checkpoint genes across elite controllers (EC), viremic controllers (VC), and chronic progressors (CP). **B** Boxplots comparing expression levels of CEACAM1, CD274, TIGIT, CD200, CTLA4, BTLA, and ADGRG1 across clinical groups. **C** Principal component analysis (PCA) based on immune checkpoint gene expression, showing distinct clustering of EC, VC, and CP samples

We investigated the expression levels of immune checkpoint genes in CD4⁺ T cells from HIV-infected individuals categorized as elite controllers (EC), viremic controllers (VC), and chronic progressors (CP). As shown in Fig. 1A and B, *CEACAM1* and *CD274* were significantly upregulated in EC compared to CP (*p* < 0.001), while VC samples exhibited intermediate expression levels for most of these genes. Conversely, significantly lower expression of *CD200*, *TIGIT*, *CTLA4*, *BTLA*, and *ADGRG1* was observed in EC compared to VC (*p* < 0.001). Principal component analysis (PCA) based on the expression of all immune checkpoint genes, revealing distinct clustering of samples according to clinical group (Fig. 1C). CP samples formed a tight cluster, clearly separated from EC samples along PC1 (23.8% of variance) and PC2 (20.5% of variance), with VC samples distributed between the two. This clustering was primarily driven by the expression of *PDCD1*, *CTLA4*, *LAG3*, and *TIGIT*, which were positively correlated with the CP group, whereas *CD226*, *SLAMF7*, and *CD28* exhibited inverse correlations, contributing to the separation of EC samples (Fig. 1C).

### Effector memory CD4⁺ T cells are enriched in elite controllers and correlate with distinct immune checkpoint profiles

**Fig. 2:**
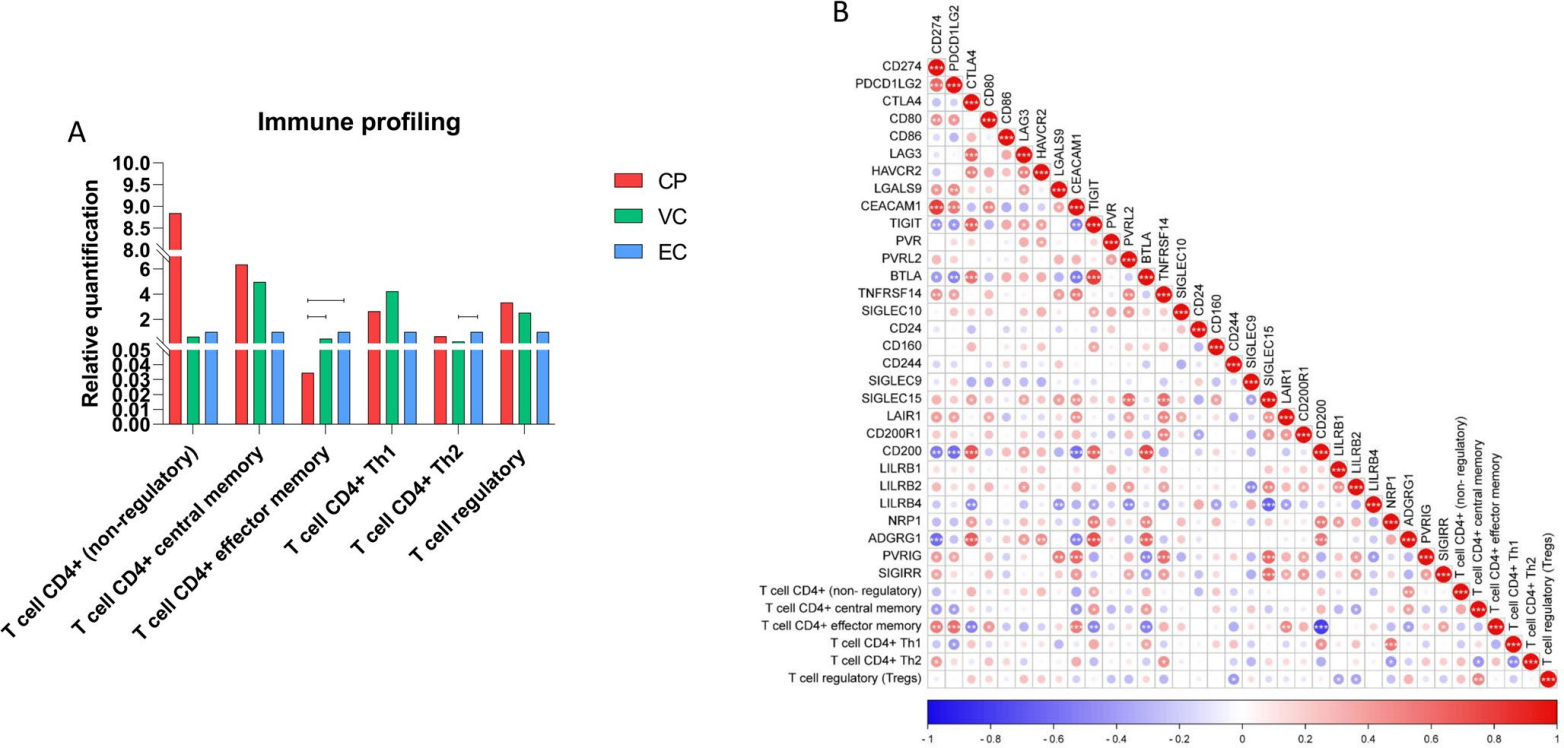
Effector memory CD4⁺ T cells are enriched in ECs and associate with specific immune checkpoint profiles. **A** Bar plots showing estimated abundance of CD4⁺ T cell subtypes across clinical groups. **B** Correlation matrix between CD4⁺ T cell subpopulations and expression of immune checkpoint genes

We next profiled the CD4⁺ T cell subpopulations in HIV-infected individuals, based on transcriptomic deconvolution analysis. As shown in Fig. 2A, ECs exhibited a significantly higher abundance of CD4⁺ effector memory T cells compared to CPs. Additionally, a significant increase in Th2 cells was observed in ECs relative to VCs. Figure 2B shows a correlation matrix between immune checkpoint molecules and the abundance of various CD4⁺ T cell subpopulations. The CD4⁺ effector memory population was positively correlated with several inhibitory checkpoint molecules, *CD274* (*PD-L1*), *PDCD1LG2* (*PD-L2*) and *CEACAM1*. In contrast, significant negative correlations were observed between effector memory T cells and *CTLA4*, *TIGIT*, *BTLA* and *CD200* (Fig. 2B).

### Identification of immune checkpoint-associated gene modules through network analysis

**Fig. 3:**
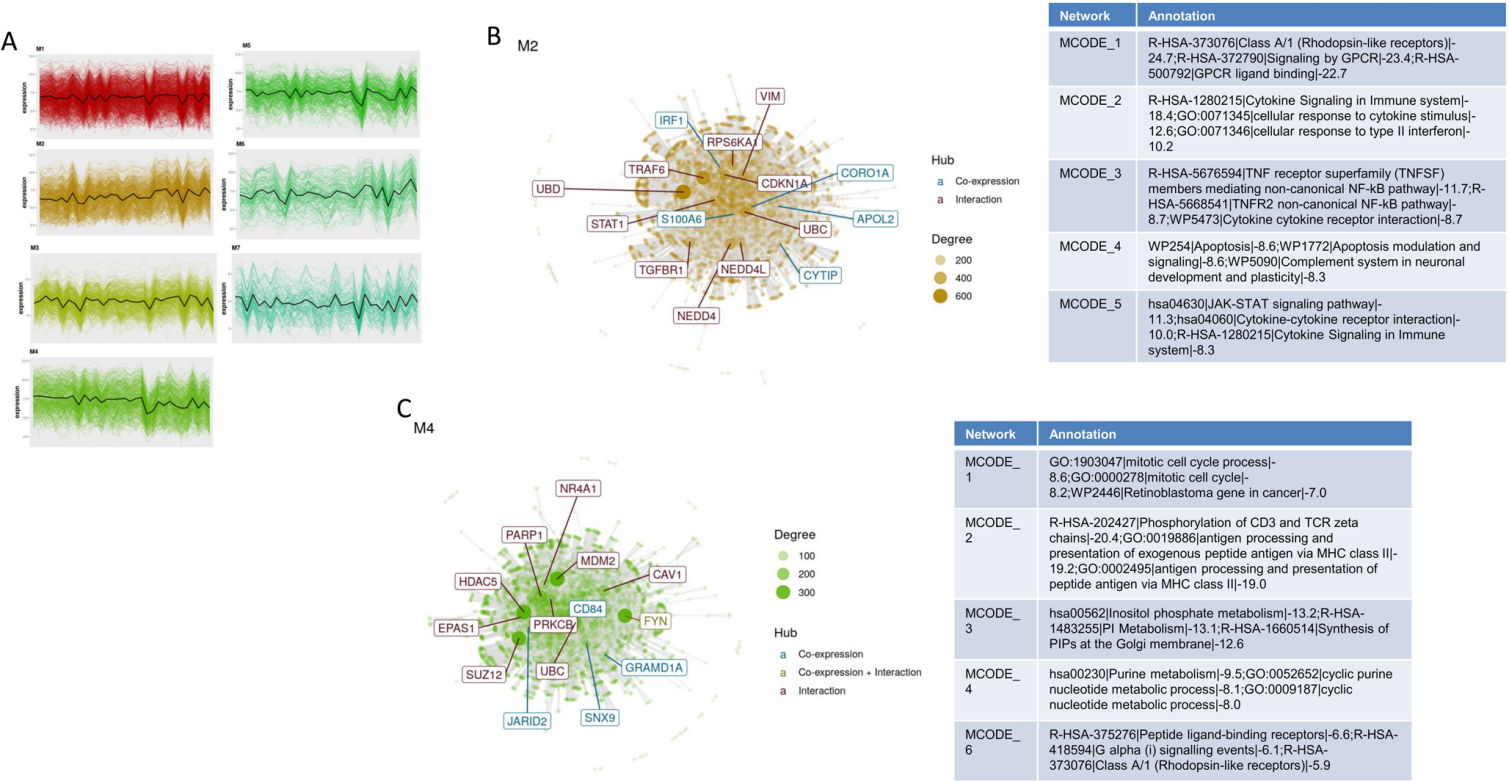
Network-based co-expression modules define immune checkpoint gene contexts. **A** CEMiTool analysis identifying seven transcriptomic modules across samples. **B** Module 2 (M2) network, including *CEACAM1* and *CD274*, with MCODE clusters and functional enrichment. **C** Module 4 (M4) network, including *TIGIT*, *BTLA*, *CD200*, and *ADGRG1*, with corresponding enriched pathways

We next performed a gene co-expression network analysis using CEMiTool on the whole-genome transcriptomic data from HIV-infected individuals. As shown in Fig. 3A, seven gene modules were detected. Module 2 (M2) includes the immune checkpoint genes *CEACAM1* and *CD274* (*PD-L1*) (Fig. 3B). MCODE clustering within M2 identified six highly interconnected sub-networks. Functional enrichment annotations show a significant involvement in immune response pathways, such as “Cytokine signaling in immune system,” “NF-κB activation,” and “Interferon signaling” (Fig. 3B). Module 4 (M4) included the immune checkpoint genes *CD200*, *BTLA*, *TIGIT*, and *ADGRG1* (Fig. 3C). MCODE analysis of M4 identified six subnetworks with strong enrichment for immune-related and metabolic pathways. These included “Mitotic cell cycle process,” “Signaling by CD3 and TCR zeta chains,” and “Purine metabolism”, among others (Fig. 3C).

### Transcriptomic comparison of elite controllers and ART-treated individuals

**Fig. 4:**
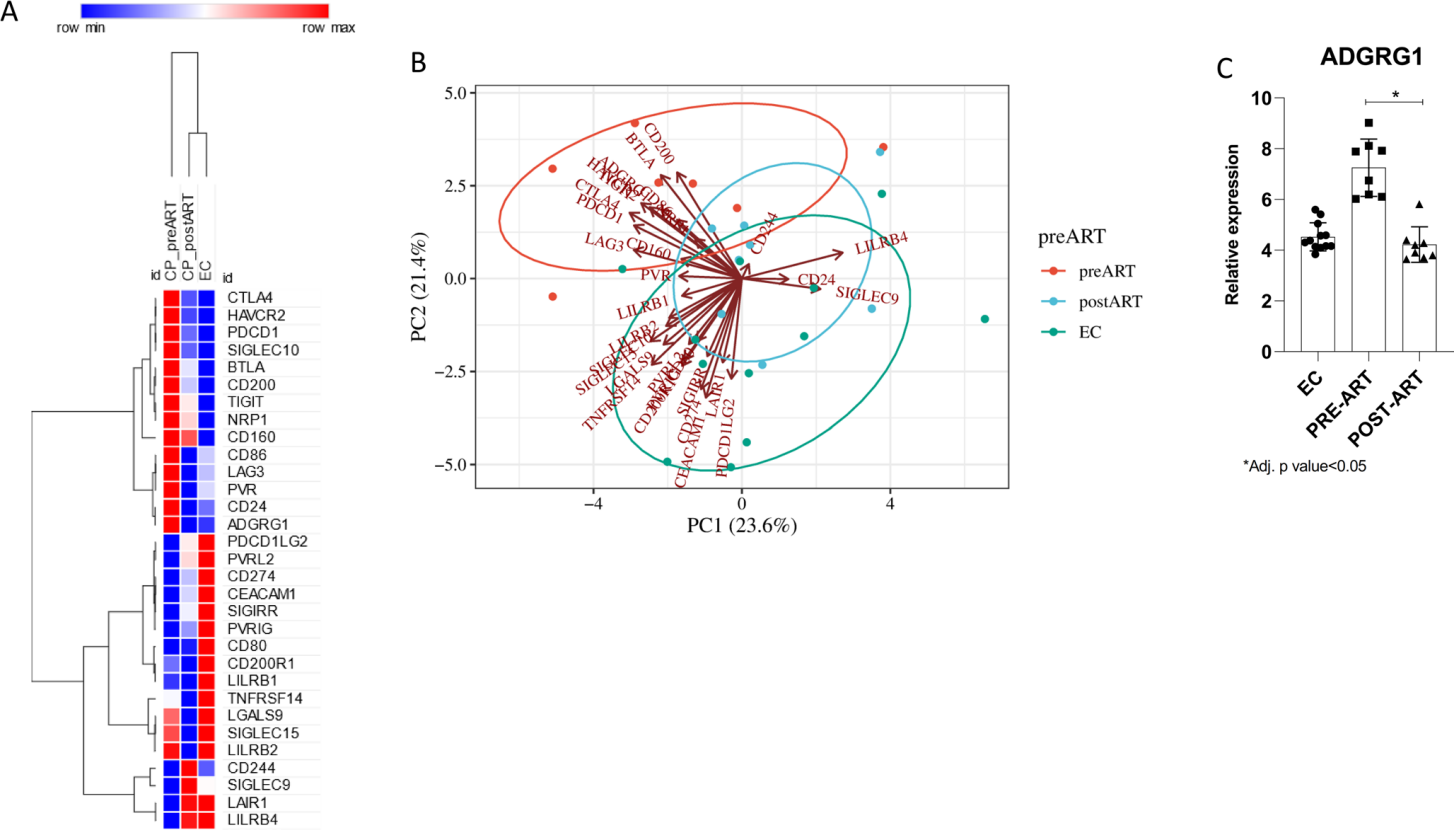
Transcriptomic comparison of immune checkpoint genes in EC, pre-ART, and post-ART individuals. **A** Heatmap of checkpoint gene expression across treatment groups. **B** PCA plot showing clustering of samples based on immune checkpoint profiles. **C** Boxplot of ADGRG1 expression across groups, highlighting significant modulation by ART

Finally, we investigated the expression of immune checkpoint genes among elite controllers (EC), pre-ART (antiretroviral therapy-naïve), and post-ART individuals. As shown in Fig. 4A distinct expression patterns between the three groups were found. Notably, ECs and post-ART individuals exhibit a closer transcriptional profile compared to pre-ART subjects, which show pronounced upregulation of several inhibitory immune checkpoints. The principal component analysis (PCA) plot indicates that pre-ART samples cluster separately from EC and post-ART groups along the principal components PC1 (23.6%) and PC2 (21.4%) (Fig. 4B). However, among the analyzed genes, *ADGRG1* (also known as *GPR56*) was identified as the only checkpoint significantly modulated across the groups (Fig. 4C), as its expression was markedly increased in pre-ART individuals compared to ECs and significantly reduced following ART initiation (*p* < 0.05), suggesting partial normalization of immune activation status.

## Discussion

Despite the transformative success of combination antiretroviral therapy (ART) in suppressing HIV replication, achieving a functional cure or complete eradication remains an unmet goal [31]. One of the central challenges is the persistence of latent viral reservoirs that evade immune clearance and reignite viremia upon treatment interruption [32]. Early studies on the kinetics of viral decay revealed that HIV persists in multiple infected cell compartments with distinct half-lives, highlighting the complexity of eliminating all replication-competent virus [33]. Even in individuals with profoundly low levels of viral reservoirs, interruption of ART leads to a rapid rebound of plasma viremia, emphasizing the robustness of these persistent reservoirs [32].

The clinical benefits of ART, including a significant reduction in HIV-related morbidity and mortality, are well documented [34]. However, ART alone does not restore full immune competence, particularly in the context of chronic immune activation and T cell exhaustion. The regulation of T cell responses is orchestrated in part by members of the B7 family, which includes both co-stimulatory and co-inhibitory ligands [35]. Among the inhibitory pathways, the PD-1/PD-L1 axis has been extensively implicated in T cell dysfunction during HIV infection. HIV-specific CD4⁺ T cells exhibit enhanced responsiveness upon PD-1 blockade, suggesting a potential for immune restoration [36]. Similarly, PD-1 expression on HIV-specific CD8⁺ T cells contributes to reversible immune dysfunction, and remains elevated in both treated and untreated individuals [36]. Notably, PD-1 signaling can be modulated by cytokines such as IL-2, which partially reverses its inhibitory effects on both CD4⁺ and CD8⁺ T cells [36].

Beyond PD-1, other inhibitory receptors such as TIGIT have gained attention as modulators of T cell function. TIGIT belongs to the CD28 family and plays a suppressive role by promoting the generation of tolerogenic dendritic cells [37, 38]. In HIV and SIV infections, TIGIT marks a subset of exhausted T cells and correlates with disease progression, making it a promising target for immune checkpoint blockade [38].

Together, these findings highlight the importance of integrating immunologic interventions with ART to achieve durable HIV remission. Strategies targeting immune exhaustion pathways, such as PD-1 or TIGIT blockade, hold promise but require further clinical investigation to optimize efficacy while minimizing immune-related adverse events.

In this study, we investigated the expression patterns of immune checkpoint molecules and the transcriptional architecture of CD4⁺ T cell subpopulations across EC, VC, and CP. Importantly, rather than focusing solely on differential expression, we applied an integrative systems immunology framework, combining dimensionality reduction, immune deconvolution, and co-expression network analysis.

This approach allowed us to uncover that immune checkpoint regulation in HIV infection is modular, context-dependent, and tightly linked to cellular differentiation states, thereby extending previous observations based on single-gene analyses.A particularly relevant observation emerging from our analysis is the identification of a non-canonical checkpoint signature in elite controllers, characterized by relatively higher expression of *CEACAM1* and *CD274* (*PD-L1*) in the context of lower expression of classical exhaustion markers. While these molecules have been previously described in immune regulation and, to a limited extent, in HIV infection, our data provide additional context by showing that they are embedded within a co-expression module enriched for interferon and NF-κB signaling pathways. This pattern suggests that their expression may be associated with regulated immune activation rather than overt dysfunction. Furthermore, their positive correlation with effector memory CD4⁺ T cell abundance indicates that these molecules may be linked to active, differentiated immune compartments, although their precise functional role cannot be established from transcriptomic data alone. Collectively, these findings support a model in which EC are characterized not by the absence of inhibitory signals, but by a balanced regulatory state in which activation and inhibition are coordinated.

Moreover, in CP, we observed a higher expression of the exhaustion markers *CD200*, *CTLA4*, *BTLA*, and *TIGIT*, which contributed prominently to the principal component–based separation of clinical groups, indicating that checkpoint-associated transcriptional variation captures relevant axes of disease heterogeneity. We note that this observation does not imply that these genes are the primary drivers of global transcriptomic differences, but rather that a biologically informed subset of immune checkpoints is sufficient to recapitulate meaningful stratification. Their coordinated expression patterns suggest the presence of shared upstream regulatory influences, potentially related to chronic antigen exposure, inflammatory signaling, and metabolic stress, although causal relationships cannot be inferred from the present data. In addition, the observed inverse association between these exhaustion-related transcripts and effector memory CD4⁺ T cell abundance supports a model in which transcriptional programs linked to dysfunction are associated with altered differentiation states, reinforcing the concept that immune impairment in HIV involves both functional and developmental components at the population level.

Another notable aspect of this study is the identification of *ADGRG1* as the only immune checkpoint consistently modulated across pre-ART, post-ART, and EC conditions. Unlike classical checkpoints, ADGRG1 is localized within a co-expression module enriched for T cell receptor signaling, cell cycle processes, and metabolic pathways, and its expression decreases following ART initiation, suggesting sensitivity to changes in antigen load and immune activation. Although these observations are associative, they point to a potentially underexplored role for ADGRG1 as a candidate marker of immune normalization rather than canonical exhaustion.

Through co-expression network analysis, we identified distinct checkpoint-containing modules with different functional enrichments, including a module associated with *CEACAM1* and *CD274* linked to interferon signaling and immune regulation, and a separate module containing *TIGIT*, *CD200*, *BTLA*, and *ADGRG1* associated with metabolic activity, proliferation, and T cell receptor signaling. We emphasize that these modules represent patterns of coordinated gene expression and do not imply direct functional interactions or causality; however, they provide a useful framework for interpreting immune checkpoint regulation as part of broader transcriptional programs rather than isolated signals. This modular perspective suggests that different aspects of immune regulation, including feedback control and exhaustion-associated processes, may be transcriptionally separable yet co-existing within the CD4⁺ T cell compartment.

By integrating transcriptomic deconvolution, we further show that EC are enriched in CD4⁺ effector memory T cells, which display positive associations with *CD274*, *PDCD1LG2*, and *CEACAM1*, and negative associations with exhaustion-related checkpoints. These findings indicate that checkpoint expression should not be interpreted as intrinsically indicative of dysfunction, but rather as reflecting the underlying activation state, differentiation status, and environmental context of the cells. Given the use of bulk transcriptomic data, these relationships likely reflect both cell-intrinsic regulation and shifts in subset composition, and therefore should be interpreted with appropriate caution.

From a translational perspective, our findings suggest that immune checkpoint biology in HIV infection may be better understood within a network framework. While it would be premature to draw direct therapeutic conclusions, these observations raise the possibility that targeting coordinated checkpoint programs, rather than individual molecules alone, could provide a more nuanced approach to immune modulation. Similarly, the association between effector memory CD4^+^ T cells and specific checkpoint patterns supports further investigation into strategies aimed at preserving or restoring functional memory compartments in combination with immunoregulatory interventions. We emphasize, however, that these implications are hypothesis-generating and require experimental validation.

A key methodological aspect of this study is the observation that a restricted panel of biologically curated immune checkpoint genes is capable of capturing meaningful variation across clinical phenotypes and recapitulating group stratification. While this does not establish these genes as the dominant drivers of global transcriptomic differences, it supports the utility of targeted, mechanism-informed gene sets for dimensionality reduction and exploratory analysis in complex immunological datasets. To strengthen the robustness of these findings, we will extend the analysis to independent cohorts and incorporate additional validation strategies.

Finally, we acknowledge several limitations of this study. The reliance on bulk CD4^+^ T cell transcriptomic data limits the resolution of cell-type-specific effects and may confound interpretation due to underlying cellular heterogeneity. In addition, the analysis is based on a single primary dataset, which may affect generalizability. Future studies integrating independent cohorts, single-cell RNA sequencing, and complementary multi-omics approaches, as well as longitudinal designs, will be essential to validate and refine the transcriptional modules identified here and to establish their functional relevance.

In conclusion, our study suggests that immune checkpoint regulation in HIV infection is best interpreted as a structured and context-dependent transcriptional landscape rather than a collection of independent inhibitory signals. EC appear to exhibit a coordinated regulatory profile consistent with balanced immune activation, whereas CP display transcriptional patterns associated with immune dysregulation. These findings provide a framework for further investigation into the systems-level organization of immune responses in HIV and may inform future studies aimed at understanding mechanisms of viral control and immune restoration.

## Data Availability

The data were generated by re-analysis of the publicly available dataset GSE128297.
